# Address Delivered before the Fellows of the Massachusetts Medical Society, at the Annual Meeting, May 28, 1851

**Published:** 1851-08

**Authors:** David Humphreys Storer


### EDITORIAL DEPARTMENT.

*Address delivered before the Fellows of the Massachusetts Medical Society, at the Annual Meeting, May* 28, 1851. By David Humphreys Storer, M. D.

The Massachusetts Medical Society enrolls among its fellows every member of the profession in good standing, within the limits of the state. In its organization, it is, therefore, in the fullest sense, popular, or, using the expression in its appropriate signification, democratic. At the annual meetings a large number of the profession are usually assembled, and after an address, and the transaction of business, the anniversary is celebrated with the festivities of a public dinner provided at the expense of the Society. For seventy years this custom has been steadily kept up. The occasion is one of no small interest, and contributes not only to promote union for common objects, but to inspire zeal and good feeling, and thus to advance the character and usefulness of the Profession. We should be glad to see a reorganization of our State Society upon a plan somewhat similar. The prosperity of the Society, and its objects, would, as we think, be better secured by such a change. This opinion, if we mistake not, is entertained by many, and we hope that, ere long, it will become the prevailing sentiment, and the reform effected. The annual meetings of the Mass. Med. Society have heretofore been held at Boston. But at the meeting preceding the last, it was resolved to assemble, on successive years, at different places within the state, and the first point selected was Worcester, where was held the annual meeting for 1851. Several hundred members were present on that occasion, a fact which presents a mortifying contrast with the result of the recent effort made by the New York State Society, to hold a semi-annual meeting in Buffalo. We blush to state that at that proposed semi-annual meeting, there were not present the requisite number of delegates to constitute a quorum! Several Physicians were present from different places who were not delegates, and those, with the few delegates who had assembled, resolved themselves into a convention, the proceedings of which will form the subject of another article.

These remarks have been suggested by the perusal of an able and interesting address by Dr. Storer, the appointed orator at the last annual meeting of the Mass. Med. Society. The subject selected by Dr. S. was "*Medical Jurisprudence, its claims to greater regard from the Student and the Physician.*" This subject is treated by Dr. S. in a practical and effective manner. Th subject is one, the importance of which is too apt to be overlooked by the Student and Physician. Occasions calling into requisition knowledge of medical jurisprudence, although they are infrequent, in comparison with the ordinary daily duties of the Profession, may happen at any moment, placing the practitioner in positions involving great responsibilities in various relations. There are, perhaps, no positions which afford better opportunities for commanding, in behalf of medical science, public respect and confidence; and, of all opportunities for this end, none, perhaps, oftener expose the profession to distrust and ridicule, owing, in a great measure, to the too general neglect of the subjects embraced in this department of medical education. Dr. Storer's address is a timely appeal, which will not only be read with interest, but will be useful in calling attention to the subject.

It is customary, in connection with the annual address before the Fellows of the Mass. Med. Society, to pay a becoming tribute to the memories of those whose connection with the Society, within the year, has been dissolved by death. In the list of those who have ended their labours on earth, is the name of an honored grandsire of the Editor of this Journal. We trust we shall be pardoned for yielding to the feeling which prompts us to transfer the obituary notice by Dr. Storer, to our columns.

"Dr. Austin Flint died at Leicester, on the 29th day of August, 1850, in the ninety-first year of his age. He was the son of Dr. Edward Flint, of Shrewsbury, where he was born, Jan. 4, 1760, and a lineal descendant, in the fourth generation, from Hon. Thomas Flint, who came from Derbyshire, in England, and settled at Concord, in this State, in the year 1638.

"Dr. Flint was an ardent patriot, and coming upon the stage of life just at the commencement of the Revolution, he joined the army as a private soldier when but seventeen years of age, was at the battle of Saratoga, and witnessed the surrender of Burgoyne's army in the fall of 1777. He subsequently served in the capacity of surgeon, for which he received a pension from the United States. Again, when the insurrection, headed by Shays, broke out in 1786, he volunteered and served as a surgeon in the detachment of militia under General Lincoln, and was present when the rebels finally fled and dispersed. He studied medicine under the direction of his father, settled at Leicester as a physician in 1783, and continued in extensive practice in that and in all the neighboring towns for about forty years, when he withdrew from practice, and devoted himself principally to agricultural and horticultural pursuits, in which he had always taken great pleasure. He continued to cultivate his garden, performing most of the work with his own hands, until within a year or two of his death.

"The honorary degree of M. D. was conferred upon him by Harvard College, in 1825.

"During the whole of the more active portion of his life, he sustained important and responsible offices in the administration of the affairs of the town, and was for many years an efficient member of the Board of Trustees of Leicester Academy. He was a representative of the town for several years in the General Court, and, after retiring from practice, was a good deal consulted and employed as a magistrate.

"His habits of life were extremely simple and unostentatious. He was always an early riser, and, during a large part of his professional life, was accustomed on horseback to visit his patients, who of course were very much scattered. As it was no unusual occurrence in those days for many of the roads in the country to be impassable, for weeks together in winter, by the fall of deep snows, he was often obliged to make his visits on foot, and not unfrequently he walked fifteen or twenty miles or more a day on snow shoes.

"His food was always plain, but substantial; and his usual beverage was cold water. He was a thorough temperance man in practice as well as profession, long before temperance societies had existence. He not only abstained from wine and strong drinks himself, but was not accustomed to place them before his friends, even at the time when general usage would have sanctioned a different course. And thus, at the age of eighty, he had as much strength and vigor as are usually found in persons who have reached the age of sixty.

"Dr. Flint was a charitable man. He not only visited and furnished medicines for the poor without fee, but contributed cheerfully his time and his money for any and all objects which were deemed worthy of patronage. Few persons in the community in which he lived, gave more than he for public purposes or in private charity; and none, probably of his limited means, gave as much as he.

"He was a Christian man in all the relations of life. He held firmly to the faith called orthodox, in which he had been educated; but there was not in him a particle of partizan zeal or of sectarian bigotry. All men were good in his eyes, who exhibited the Christian life in their daily conversation.

"With such habits and feelings, he continued to enjoy life to an extreme old age himself, and to be an object of friendly interest and regard, not only to his family, but to all who had the pleasure of his acquaintance, even to the close of a life of fourscore years and ten."

